# It Is Not the Virus Exposure: Differentiating Job Demands and Resources That Account for Distress during the COVID-19 Pandemic among Health Sector Workers

**DOI:** 10.3390/ijerph20021212

**Published:** 2023-01-10

**Authors:** Alejandra del Carmen Dominguez-Espinosa, Johnny R. J. Fontaine

**Affiliations:** 1 Psychology Department, Ibero-American University, Mexico City 01219, Mexico; 2 Department of Work, Organization and Society, Faculty of Psychology and Educational Sciences, Ghent University, 9000 Ghent, Belgium

**Keywords:** COVID-19 traumatic stress, burnout, generalised anxiety, depression, health-sector workers, virus exposure

## Abstract

A cross-sectional study of 3860 health-sector workers across two data collections was conducted to identify the predictive power of different job demands and job resources during the COVID-19 pandemic based on four indicators of distress (COVID-19 traumatic stress, burnout, generalised anxiety, and depression) among health-sector workers. Exploratory and confirmatory factor analyses, measurement invariance checks, and structural equation models were used to evaluate the dimensionality and the effect of the job demands and resources on distress indictors. The identified job demands were workload, confinement, loss, and virus exposure, while the identified job resources were self-efficacy, momentary recuperation, and meaning making. Loss and workload predicted the distress indicators best, while confinement and virus exposure mainly predicted COVID-19 traumatic stress and were less important for the other distress outcomes. Self-efficacy and meaning making negatively predicted distress, while momentary recuperation, controlled for the other demands and resources, was positively related to the distress indicators. Of the typical pandemic-related demands and resources, the experience of loss due to COVID-19 infection was the most important predictor of distress outcomes. Confinement, and especially the awareness of virus exposure, were far less important predictors.

## 1. Introduction

In the last two years, difficulties in the work environment increased due to emerging stressors linked directly to the COVID-19 pandemic, such as shortage of protective equipment [1] and fear of infection [2], which led to higher levels of burnout, stress, anxiety, and depression [3,4,5], primarily affecting health workers [6]. As new stressors emerged during the pandemic, it is important to identify their specific role in work-related distress. Using the Job Demands and Resources model JR-D [7,8], which has proven its effectiveness in predicting burnout and other distress reactions in the work environment [9,10], the present work aimed to identify the specific demands and resources required during the COVID-19 pandemic, and their links with distress outcomes among health-sector workers. Mexico was one of the countries with the highest numbers of COVID-19 cases; it was only surpassed by the U.S. and Brazil [11]. Seventy percent of deaths caused by COVID-19 occurred in the public health system [5,12].

According to the JD-R model [7,8] any job demand (physical, psychological, social, or organisational) required to perform a job, can evolve into a stressor that does not allow the employee to recover from the emotional or physical drain caused by such demands. On the other hand, there are job resources (physical, psychological, social, or organisational) that help the person to functionally cope with the job demands and their associated costs [13].

The relationship of job demand with weakening of people’s mental health was established long before COVID-19, e.g., [14,15]. The constant exposure to job demands can lead to experiences of burnout [13,16,17]; emotional problems, such as anxiety and depression [18,19,20,21,22]; substances use [23,24,25]; adverse work-related outcomes [26]; and a higher risk of mortality [27]. In contrast, job resources refer to those factors that facilitate achievement of work-related goals, help reduce job demands, and increase work engagement [28]. Job resources have been shown to reduce burnout and other psychological problems [29,30,31].

The healthcare sector suffered the direct impact of the COVID-19 pandemic-related job demands the most. Physicians [30,32], nurses [33,34], pharmacists [35,36], and janitors working at hospitals or clinics [37] were among the affected healthcare professions. The health workers’ mental health was affected [6,25] with increased levels of burnout [38], traumatic stress [39], anxiety [40], and depression [41]. In Mexico, the sense of unpreparedness, lack of protective equipment and sanitary rules, and an overwhelmed health system have contributed to physical and mental breakdowns of health workers, resulting in a decrease in cognitive abilities such as attention, understanding, and decision making [5], as well as insomnia, depression, and post-traumatic stress syndrome, mainly associated with loss and workload [6].

In the early stages of the COVID-19 pandemic, Balasubramanian et al. [42] identified ten stressors, or job demands, that affect health workers, such as high viral load exposure, fear of getting sick of COVID-19 and infecting others (also referred to as COVID traumatic stress), grief for loss of known and unknown persons, and increase in workload. These authors also identified individual and workplace positive measures—job resources—that could help health professionals to cope with stressors. The list of individual job resources included, among others, retreating to a private space for a moment, speaking to a close friend, and getting regular exercise. The list of workplace job resources included five positive measures, such as having a workplan for the day and using personal protective equipment.

COVID-19 traumatic stress takes a specific position among the stressors that are specific to the COVID-19 pandemic, as identified by Balasubramanian et al. [42]. It forms the most direct psychological distress reaction to COVID-specific job demands and can also negatively affect mental well-being. Therefore, COVID-19 traumatic stress is included in the current study as a mediator between the job demands and resources on the one hand, and general distress outcomes on the other hand. Thus, the general expectations of the current study are that job demands (negatively) and job resources (positively) affect mental health [9], and achieve this especially through the experience of COVID traumatic stress. Furthermore, the contribution of specific COVID-related job demands and job resources to distress outcomes is investigated exploratively.

## 2. Materials and Methods

A cross-sectional study was conducted by means of an online survey across two data collections during the second and third COVID-19 waves in Mexico. The survey was part of a pilot intervention program which aimed to ameliorate the potential distress suffered by hospital workers during the COVID-19 pandemic. Participation in the survey was completely voluntary, anonymous, and required the participant to give his/her informed consent before enrolling. 

### 2.1. Participants

The first data collection consisted of a total of 1259 registered participants recruited between June and July of 2021. All the participants were health workers affiliated with the Mexico City Ministry of Health. After applying standard procedures to delete cases with missing data in the sociodemographic section or incomplete records in the psychological scales sections, a final sample size of 1014 valid cases remained. 

For the second round of data collection, 3453 Mexican health sector workers enrolled in the Mexican Social Security Institute Labor Union completed the survey. After eliminating cases without available data in sociodemographic and psychological scales, a total of 2846 records remained. 

### 2.2. Instruments

The full survey consisted of nine scales. The present paper focuses on five scales, presented in the same order to every participant across six sections. The first section consisted of sociodemographic questions, and the five other sections contained the following scales: 

Burnout Scale. The short version of the Mexican Burnout Scale [43] was adapted to a hospital context. It consisted of 12 items operationalising the three dimensions of burnout of Maslach and Jackson’s process model [44]: Emotional exhaustion (5 items, e.g., I have a hard time getting up in the morning to go to work), depersonalisation (3 items, e.g., I have found that the people I serve respect me more if I treat them badly), and achievement dissatisfaction (4 items, e.g., My work activities no longer seem important to me). A global index of occupational burnout can be obtained by summarising all 12 items. The internal consistencies (Cronbach’s alpha) were 0.87, 0.83, and 0.83, and for the second round of data collection, they were 0.86, 0.85, and 0.86 for the three dimensions, respectively.

COVID-19 Traumatic Stress Scale. This scale was an adaptation of the COVID-19 Stress Scale (C19-TS) by Delgado-Gallegos et al. [5] to the hospital context. For the first round of data collection, the version consisted of six items, and for the second round of data collection, one additional item was included (e.g., I had bad dreams about viruses). Cronbach’s alphas were 0.84 and 0.90 in the first and the second data collection, respectively.

Generalised Anxiety. The scale for Generalised Anxiety Disorder (GAD) proposed by Spitzer et al. [45] and translated into Spanish, consisted of seven items (e.g., Did you feel it was difficult to relax?). Cronbach’s alphas were 0.92 for both data collections.

Depression Scale. The Spanish version by Franco-Díaz et al. [46] of the Depression Scale (Center for Epidemiological Studies Depression Scale; CESD), consisted of seven items in a question format (e.g., did you feel sad?). Evidence of one-dimensionality was obtained from an exploratory factor analysis with an explained variance of 57%. In the present study, one item was deleted (did you sleep well?) as its content was semantically too closely linked to emotional exhaustion in the burnout scale. Cronbach’s alphas were 0.90 for both data collections.

Job demand and resources instrument. The stressors identified by Balasubramanian et al. [42] during the first wave of the COVID-19 pandemic formed the primary source of information to develop a self-report instrument for COVID-related job demands and job resources framed within the JD-R model [8,13]. The stressors were rephrased as declarative statements in the first person. There were 11 and 13 job demands items (henceforth named JDI), and 13 job resources items (henceforth named JRI). 

The five scales used a response scale with four response options ranging from never (0) to all the time (4) over the last two weeks. The language of the survey was Mexican Spanish.

### 2.3. Procedure

Both data collections were carried out via online resources using the Research Electronic Data Capture REDCap 10.6.11 [47] with a non-probabilistic sampling procedure. Healthcare workers were invited to participate in the current study within a framework of an online psychoeducational intervention program. This program consisted of a series of short courses concerning tools for coping with distress. The invitation to participate in this program was sent via email and announced within intranet mail services. The authors of the manuscript did not have access to the email lists. The people in charge of sending the invitation were part of the health institution’s staff.

The program was offered at no cost, but registration was necessary to access the content. Before the videos of the courses were presented, a pop-up window asked if the person would be interested in participating in a survey. If the person agreed to participate, informed consent was displayed. After the person accepted the informed consent, the survey started. The survey could be paused or stopped at any time, and the participant had the option to go directly to the online courses instead. When participants finalised the survey, they received feedback on their scores. If their scores were high, they were encouraged to act and get in contact with psychological services. Participants were warned against using the survey as a self-diagnosis tool; they were rather advised to use it as guideline to seek professional support. Participants were redirected to the online courses after finalising the survey. The survey was assembled to last approximately 25 min. 

### 2.4. Data Analyses

To avoid spurious relationships between the demands and resources, on the one hand, and the distress outcomes, on the other hand, because of overlap in the item content (problem of tautology), nine items were removed before starting the analyses, e.g., [48,49].

The first dataset was used as an exploratory dataset to identify the dimensional structure of the job demands and the job resources. The most parsimonious structures were identified using Principal Component Analyses. After identifying these dimensional structures, a series of confirmatory factor analyses were conducted first on the exploratory dataset and then independently on the second, confirmatory dataset. Potential differences between both datasets were investigated using measurement invariance tests.

Finally, after combining the two datasets, a structural equation (path) model with latent variables was run, in which the job demands and job resources are predictors for COVID-19 traumatic stress, burnout, generalised anxiety, and depression, and COVID-19 traumatic stress functioning also acted as a mediator.

## 3. Results

Of the first data collection, seven hundred and ninety-one participants identified themselves as female (78%); 223 as men (22%); 585 as single or divorced (58%); 429 as married or in free union (42%); with an age range from 19 to 81 years old (M_age_ = 37.36 years; S.D. _age_ = 11.32 years); with secondary level of education (years 7 to 12; 6%), bachelors’ diploma (56%) and postgraduate studies (medical specialisation, masters’ and doctoral’ degrees, 38%); working in clinical and medical care (59%), administrative position (16%), clinical and laboratory analyses (0.5%), and other areas (24.5%). The descriptive results of the second data collection indicated that two thousand and ninety-nine participants identified themselves as female (74%); 747 as men (26%); 1314 as single or divorced (46%); 1531 as married or in free union (54%); with an age range from 18 to 86 years old (M_age_ = 38.49 years; S.D. _age_ = 9.07 years); with secondary level of education (years 7 to 12; 26.5%), bachelors’ diploma (44%), and postgraduate studies (medical specialisation, masters’ and doctoral’ degrees, 29.5%); working in clinical and medical care (52.4%), administrative positions (19.7%), clinical and laboratory analyses (2.2%), and other areas (25.7%). A series of Principal Component Analyses (PCAs) using the first database were run on the JDI to exploratively investigate their dimensionality. One up to four-dimensional representation was explored. In the one-dimensional PCA solution, all factor loadings were higher than 0.40; however, the communalities were rather low. As the numbers of dimensions increased, the factorial solution became conceptually clearer, communalities increased, and the minimum factor loading reached 0.81 with no cross-loadings. These four dimensions were labelled as workload (item 7. I have been assigned tasks of more responsibility; item 11. My workload has increased), confinement (item 9. I have lost freedom of mobility to spaces or places within work; item 10. I have had fewer opportunities to carry out leisure or distraction activities at work), loss (item 5. I have gone through periods of grief due to the loss of a family member or friend; item 6. I have experienced periods of grief due to the loss of a patient or colleague at work), and virus exposure (item 1. I have been exposed to a strong viral load in the work environment; item 2. I have had difficulty protecting myself through equipment or clothing [e.g., face shield, face mask, antibacterial gel] during my work hours). Because of its conceptual clarity, the four-dimensional solution was selected.

The second set of PCAs on the JRI used the same approach. A one- up to a four-dimensional solution was explored. The single-dimension solution showed minimum factor loadings of 0.40 for all items. The three-dimensional solution was conceptually the most interpretable. Three items showed cross-loadings in the three-dimensional structure. As they were also conceptually referring to more than one dimension, these three items were deleted from the subsequent analyses. The three dimensions were labelled as self-efficacy (item 11. I have felt able to face problems at work; item 12. I think I can put work problems in their proper dimension; item 13. I have felt capable of finding solutions to problems in my work), momentary recuperation (item 1. I have been able to withdraw to a private space for a moment when I have needed it, at work; item 2. I have been able to talk about things with a close and reliable friend at work), and meaning making (item 6. I have practiced breathing techniques or meditation, inside and outside of work; item 8. I can resort to prayer according to my beliefs, whether at work or outside of it).

To corroborate the dimensionality of the JDI and JRI, we ran a confirmatory factor analysis with parameter constrains using the maximum likelihood estimator, which was performed on each dataset separately. We included the items of the outcome variables as well, each loading on their respective factor to check for discriminant evidence (see Supplemental Material). For the first dataset, the fit measures were χ^2^(832) = 2095.791, *p* < 0.001; CMIN = 2.51; CFI = 0.947; TLI = 0.940, RMSEA = 0.039 [0.037–0.041]; SRMR = 0.036; the fit measures for the second dataset were χ^2^(832) = 4258.292, *p* < 0.001; CMIN = 5.11; CFI = 0.954; TLI = 0.947, RMSEA = 0.038 [0.037–0.039]; SRMR= 0.036. The chi-square tests were significant due to the large sample sizes. However, the fit statistics (CFI and TLI) were higher than 0.94, and the misfits statistics (RMSEA and SRMR) were lower than 0.04 in both datasets. The model thus showed a fair to good fit, according to the currently suggested cut-off criteria [50]. Moreover, the CMIN criterion suggested reasonable fit [51].

To investigate whether both datasets could be merged into one large dataset, measurement invariance was investigated [52]. We examined five increasingly restrictive measurement invariance models: configural (equal factor models), metric (equal factor loadings), scalar (equal intercepts), strict (equal measurement errors), and strict plus (equal factor covariances). All levels of invariance yielded fit indexes above what is considered acceptable to good fit. The strict plus invariance model, the most restrictive model where all constrains (factor loading, intercepts, error, and factor covariances) are set to be equal across groups, was supported (Table 1). Subsequently, we merged the two datasets into one large dataset.

First, the bivariate correlations of the latent job demands and job resources with the latent distress indicators were explored (Table 2). As expected, all job demands were positively and all job resources were negatively related to the distress outcomes, including COVID-19 traumatic stress. Moreover, COVID-19 traumatic stress related strongly to the other distress outcomes.

**Figure 1:**
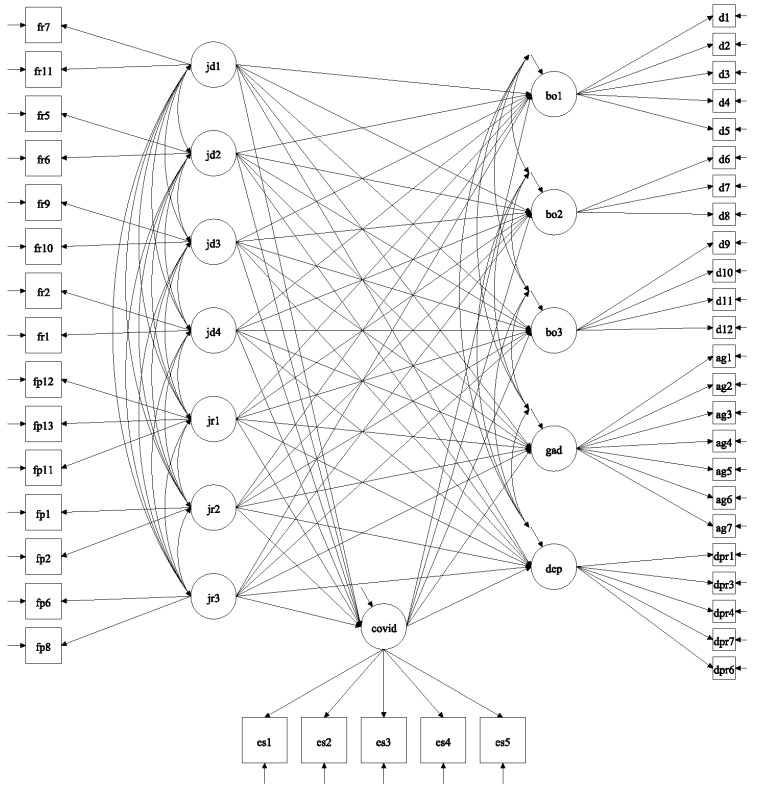
Structural equation path model of job demands and resource on distress outcomes with COVID-19 traumatic stress as mediator. Note: Latent constructs are shown in ellipses, observable variables are shown in rectangles, measurement error terms are not shown. fr1 to fr11 = job demands items; fp1 to fp13 = job resources items; jd1= workload; jd2 = confinement; jd3 = loss; jd4 = virus exposure; jr1 = self-efficacy; jr2 = momentary recuperation; jr3 = meaning making; covid = COVID-19 traumatic stress; bo1 = emotional exhaustion; bo2 = depersonalisation; bo3 = achievement dissatisfaction; gad = generalised anxiety; dep = depression; cs1 to cs5 = COVID-19 traumatic stress items; d1 to d12 = burnout items; ag1 to ag7 = generalised anxiety items; dpr1 to dpr6 = depression items. Double-headed arrows represent covariances. Single arrows represent direct effects.

To investigate the specific impact of the specific job demands and resources, we subsequently tested a structural equation path model with COVID-19 traumatic stress as a mediator between the job demands and resources on one hand, and the mental well-being outcomes on the other hand (Figure 1). This path model fitted the data well: χ^2^(832) = 5415.464, *p* < 0.001; CMIN = 6.50; CFI = 0.953; TLI = 0.947, RMSEA0.038 [0.037–0.039]; SRMR= 0.036 (Table 3). 

Confinement, loss, and virus exposure were positive predictors of COVID-19 traumatic stress, while workload and self-efficacy were negative predictors. With respect to emotional exhaustion, COVID-19 traumatic stress, workload, loss, and momentary recuperation were positive predictors, while meaning making was a negative predictor. Depersonalisation was significantly predicted by COVID-19 traumatic stress, workload, loss, and momentary recuperation, while self-efficacy and meaning making had the opposite effect. COVID-19 traumatic stress, workload, loss, and virus exposure positively predicted achievement dissatisfaction, while confinement and self-efficacy acted as negative predictors. For generalised anxiety and for depression, all predictors were significant (except confinement for general anxiety). Something to highlight here is momentary recuperation was positively related, while virus exposure was negatively related to general anxiety and depression. Thus, when all demands and resources, as well as COVID-19 traumatic stress, were considered, the relationships of momentary recuperation and virus exposure with general anxiety and depression were reversed (compared to the bivariate correlations). 

## 4. Discussion

The findings supported the two main premises of the study: job demands, also those that are highly specific for a pandemic context, have positive relationships with distress outcomes, and job resources have negative relationships with distress outcomes. These results are consistent with previous findings [6,9,36].

More interesting are the detailed analyses of the job demands and job resources. When jointly analysing their predictive relationships with distress indicators, including COVID-19 traumatic stress as a mediator, the relationship of some job demands and job resources became non-significant, or even had the opposite sign. 

COVID-19 traumatic stress functioned as a mediator [53]. It was predicted by five of the seven job demands and resources and predicted all distress outcomes. Interestingly, it predicted general mental well-being (depression and general anxiety) better than job-related mental well-being (burnout).

Of all job demands and resources, the experience of loss was the most important predictor of distress. It was the strongest predictor of COVID-19 traumatic stress, and positively predicted all five distress outcomes over and above the predictive value of COVID-19 traumatic stress. Such relationships have been reported previously, as the likelihood of experiencing periods of grief due to the loss of a patient or colleague at work are higher in the health sector than any other work setting [39]. The present study also points to the centrality of the loss experience during the COVID-19 pandemic.

Even though confinement was identified as a recurring stressor during the COVID-19 pandemic due to the strong mobility restrictions [37,38], its role turned out to be much more limited and indirect. Together with loss, it was a good predictor of COVID-19 traumatic stress, but there was by and large no additional predictive value for distress outcomes (except for depression). Confinement is probably a double-edged sword during a pandemic. It causes depression, but also prevents suffering, making its specific impact less straightforward.

Surprisingly, the awareness of virus exposure—which is at the source of the pandemic—did not play a major role in predicting distress. It had a small predictive value for COVID-19 traumatic stress (and much less important than the experience of loss and confinement) and had inconsistent relationships with the distress outcomes when COVID-19 traumatic stress was controlled for (a small positive relation with achievement dissatisfaction, no relationships with emotional exhaustion and depersonalisation, and a *negative* relationship with generalised anxiety and depression). This finding is the more surprising, as health workers were more exposed to viral load than any other professional sector, and moreover experienced a limited supply of protective equipment [36]. Possibly, as exposure to virus itself cannot be directly observed (one can never be sure of having been exposed to the virus), but only derived from what is happening around the person (such as patients and colleagues falling ill or even passing away and from restrictions due to confinement), it is mainly the consequences of the virus that cause the psychological distress, rather than the awareness of the virus itself.

While workload positively predicted all distress outcomes, and especially emotional exhaustion, which is in line with the literature [3,54], another surprising finding was that it negatively related to COVID-19 traumatic stress. A possible explanation could be that people who had a higher workload had less time to ruminate about the possible consequences of a COVID-19 infection. 

When looking at the job resources, the results were mixed. While the bivariate correlations were negative with all distress outcomes, when studying their role jointly with the job demands, in the path model, only small negative up to even small positive relationships were observed. Moreover, job resources did not predict COVID-19 traumatic stress. 

In line with the literature showing that self-efficacy can form a buffer between job demands and distress [55,56], negative, but small, relationships with COVID-19 traumatic stress and some distress outcomes were observed.

Meaning making clearly emerged as a buffer, especially for emotional exhaustion and depression. By reappraising and seeking a more positive view of the situation, the impact of a stressful situation can decrease. More specifically, praying is a coping strategy used to face adversity and ambiguous and unescapable situation, as people make use of their own beliefs to make sense of the situation [57]. Additionally, physiological relaxation has proven to be effective at reducing stress [54].

Momentary recuperation, which is assumed to be a job resource, was either not or positively associated with distress outcomes in the path model. It has been suggested in the literature that talking to a reliable friend or having time out at work can reduce stress levels [42]. Possibly, momentary recuperation can also function as a double-edged sword. According to previous research, being constantly exposed to news about the virus can lead to an increase in burnout levels [58]. If we consider that health workers were constantly exposed to (un)official news about the COVID-19 pandemic in their workplace, and this information was also shared and discussed with colleagues and close friends at work, a circle of constant exposure to information could have been created. When controlling for more constructive job resources, this constant exposure could lead to emotional exhaustion, depersonalisation, anxiety, and depression, as health workers can share distressing descriptions of COVID-19 experiences with other colleagues within the same job environment [59,60]. Self-isolation for brief periods of time at work could have immediate positive effects, as suggested previously [42]; however, over a longer period, this short-term strategy may not produce a long-term benefit as it is seen as a passive acceptance strategy [61], postponing the decision to ask for professional counselling or psychotherapeutic assistance.

The differences between the bivariate correlations and the relationships in the path model made clear that the role of job demands and resources needs to be studied in relation to one another within the context in which they occur.

A strength of the current study is that it consisted of two large data collections within the same Mexican health sector collected through two different channels. This allowed us to follow a combined exploratory confirmatory analysis strategy. In the first exploratory sample, a well-interpretable model could be identified, which was then tested in a second confirmatory sample. Moreover, measurement invariance tests showed that not only was the same factor model applied to both samples, but that also the size of the parameters was by and large the same, which supports the replicability and robustness of the current results. Moreover, by using structural equation modelling, latent relationships could be investigated, which are statistically controlled for unreliability. 

A limitation of the current study was that random sampling procedures were not used, which limits the generalisability of the findings. All the participants voluntarily agreed to complete the survey, which may have led to a self-selection bias. Moreover, the survey was distributed online, which reduced the likelihood that participants with restricted access to electronic infrastructure could participate (e.g., janitors).

Another limitation of the current study is that it was only conducted in Mexico. To what extent the relative importance of the different job demands, and resources can be generalised to other countries remains to be demonstrated. It is, for instance, very possible that the centrality of the loss experiences in predicting the outcomes is due to the very high death toll of the COVID-19 pandemic in Mexico compared to other countries.

The insights of the present research can be translated into practical organisation-directed interventions. Based on the current results, these interventions should especially focus on the reduction of workload (e.g., through schedule changes, such as shift rotation); on creating a safe space in moments of loss by enhanced communication between organisational members [62], and by fostering meaning making. Having a better view about which specific job demands and resources relate to which distress outcomes can result in better organisational interventions, which can help workers to cope better with emerging stressors in emergency scenarios.

## 5. Conclusions

The present research demonstrates that job demands and resources are not created equal. Some mainly contribute to distress through their relationship with COVID-19 traumatic stress (e.g., confinement); others directly relate to distress outcomes independent of COVID-19 traumatic stress (e.g., meaning making), while still others consistently affect both COVID-19 traumatic stress and outcomes (e.g., loss). Moreover, obvious sources of distress during the COVID pandemic, such as the awareness of virus exposure, only have a marginal contribution. To understand the role of job demands and job resources, it is important to study them within the specific context in which they operate. It is necessary to empirically investigate rather than assume the extent to which the general findings of the JD-R model apply to work situations characterised by an emergency, as was the case during the COVID-19 pandemic.

## Figures and Tables

**Table 1:** Measurement invariance across the two datasets.

MODEL	χ^2^	df	CMIN	CFI	TLI	RMSEA (LB–UB 90%)	SRMR
1st data base	2101.69	832	2.53	0.947	0.940	0.039 [0.037–0.041]	0.042
2nd data base	4258.29	832	5.12	0.954	0.947	0.038 [0.037–0.039]	0.036
Configural invariance	6359.98	1664	3.82	0.952	0.945	0.038 [0.037–0.039]	0.038
Metric invariance	6404.72	1687	3.80	0.952	0.946	0.038 [0.037–0.039]	0.038
Scalar invariance	6727.53	1718	3.92	0.949	0.944	0.039 [0.038–0.040]	0.039
Strict invariance	6913.09	1762	3.92	0.947	0.944	0.039 [0.038–0.040]	0.039
Strict plus invariance	7141.79	1840	3.88	0.946	0.944	0.039 [0.038–0.040]	0.047

Note: CMIN = Chi-Square value, CFI = Comparative Fit Index; TLI = Tucker–Lewis Index; RMSEA = Root Mean Square Error of Approximation; SRMR = Standardised Root Mean Squared Residual.

**Table 2:** Bivariate correlations of the latent job demands and job resources with the latent distress indicators.

	COVID-19 Traumatic Stress	Emotional Exhaustion	Depersonalisation	Achievement Dissatisfaction	Generalised Anxiety	Depression
COVID-19 Traumatic Stress	-	0.56	0.44	0.43	0.74	0.67
Job Demands						
Workload	0.38	0.65	0.39	0.51	0.50	0.43
Confinement	0.57	0.40	0.27	0.30	0.46	0.53
Loss	0.60	0.64	0.46	0.54	0.62	0.58
Virus exposure	0.51	0.58	0.37	0.49	0.49	0.44
Job Resources						
Self-efficacy	−0.28	−0.33	−0.31	−0.31	−0.36	−0.34
Momentary recuperation	−0.18	−0.20	−0.16	−0.21	−0.19	−0.20
Meaning making	−0.21	−0.40	−0.29	−0.30	−0.33	−0.32

Note: All correlations were significant at *p <* 0.01.

**Table 3:** Standardised relationships of job demand and resources with mental wellbeing outcomes and COVID-19 traumatic stress as mediator in structural equation path model.

	COVID-19 Traumatic Stress	Emotional Exhaustion	Depersonalisation	Achievement Dissatisfaction	Generalised Anxiety	Depression
Mediator						
COVID-19 traumatic stress	-	0.27 ***	0.24 ***	0.15 ***	0.56 ***	0.42 ***
Job demands						
Workload	−0.12 ***	0.38 ***	0.16 ***	0.23 ***	0.18 ***	0.08 **
Confinement	0.32 ***	−0.01	−0.04	−0.07 *	0.01	0.21 ***
Loss	0.37 ***	0.14 ***	0.20 ***	0.20 ***	0.18 ***	0.19 ***
Virus exposure	0.15 ***	0.05	−0.03	0.12 **	−0.10 **	−0.12 **
Job resources						
Self-efficacy	−0.12 ***	−0.03	−0.12 ***	−0.12 ***	−0.07 **	−0.06 *
Momentary recuperation	0.02	0.17 ***	0.12 **	0.01	0.14 ***	0.13 ***
Meaning making	−0.03	−0.32 ***	−0.18 **	−0.08	−0.22 ***	−0.24 ***

Note. *** *p* < 0.001, ** *p* < 0.01, * *p* < 0.05.

## Data Availability

The data file can be downloaded at: https://doi.org/10.6084/m9.figshare.21280461.v1.

## Supplementary Materials

The following supporting information can be downloaded at: https://www.mdpi.com/article/10.3390/ijerph20021212/s1, Table S1: Principal Component Analyses on the Job Demands Items; Table S2: Principal Component Analyses on the Job Resources Items; Table S3: Factor loading per item per latent factor.
