# Supplementary material for: It Is Not the Virus Exposure: Differentiating Job Demands and Resources That Account for Distress during the COVID-19 Pandemic among Health Sector Workers
Source: Int J Environ Res Public Health. 2023 Jan 10;20(2):1212. doi: 10.3390/ijerph20021212 (PMC9859370; doi:10.3390/ijerph20021212)
Supplement: Supplementary file 1 [file ijerph-20-01212-s001.zip › ijerph-2013096-supplementary.pdf]

Table S1. Principal Component Analyses on the Job Demands Items.

| Items | $h^2$ | Λ | $h^2$ | Λ | | $h^2$ | Λ | | | $h^2$ | Λ | | | |
|---|---|---|---|---|---|---|---|---|---|---|---|---|---|---|
| | | **1** | | **1** | **2** | | **1** | **2** | **3** | | **1** | **2** | **3** | **4** |
| 1. I have been exposed to a strong viral load in the work environment. | .38 | .61 | .39 | **.59** | .09 | .69 | .09 | -.04 | **.80** | .72 | .15 | -.05 | .01 | **.81** |
| 2. I have had difficulty protecting myself through equipment or clothing (e.g., face shield, face mask, antibacterial gel) during my work hours. | .29 | .54 | .29 | **.48** | .15 | .77 | -.10 | -.02 | **.92** | .77 | -.13 | .07 | -.02 | **.89** |
| 5. I have gone through periods of grief due to the loss of a family member or friend. | .18 | .43 | .77 | -.11 | **.91** | .78 | -.12 | **.93** | -.05 | .79 | -.09 | .01 | **.91** | -.04 |

| | | | | | | | | | | | | | | | |
|---|---|---|---|---|---|---|---|---|---|---|---|---|---|---|---|
| 6. I have experienced periods of grief due to the loss of a patient or colleague at work. | .34 | .58 | .70 | .12 | **.79** | .72 | .09 | **.82** | .01 | .75 | .12 | .01 | **.82** | .03 |
| 7. I have been assigned tasks of more responsibility. | .43 | .65 | .55 | **.77** | -.14 | .62 | **.83** | -.08 | -.05 | .83 | **.94** | -.13 | .05 | .03 |
| 9. I have lost freedom of mobility to spaces or places within work. | .50 | .71 | .50 | **.59** | .25 | .52 | **.53** | .28 | .10 | .74 | -.03 | **.83** | .12 | .01 |
| 10. I have had fewer opportunities to carry out leisure or distraction activities at work. | .49 | .70 | .52 | **.71** | .05 | .55 | **.65** | .08 | .11 | .80 | .04 | **.89** | -.08 | .01 |
| 11. My workload has increased. | .48 | .70 | .64 | **.84** | -.18 | .75 | **.92** | -.11 | -.09 | .79 | **.81** | .20 | -.06 | -.05 |

| | | | | |
|---|---|---|---|---|
| Proportion Total Explained | | | | |
| Variance | .39 | .55 | .67 | .77 |

*Note:* $h^2$= Communality; Λ= Factor loadings; Boldface indicates higher factor loading.

Table S2. Principal Component Analyses on the Job Resources Items.

| Items | **h²** | **Λ** | **h²** | **Λ** | | **h²** | **Λ** | | | **h²** | **Λ** | | | |
|---|---|---|---|---|---|---|---|---|---|---|---|---|---|---|
| | | **1** | | **1** | **2** | | **1** | **2** | **3** | | **1** | **2** | **3** | **4** |
| 1. I have been able to withdraw to a private space for a moment when I have needed it, at work. | .34 | .59 | .44 | .07 | **.63** | .62 | .04 | -.03 | **.79** | .64 | .06 | **.74** | .05 | .05 |
| 2. I have been able to talk about things with a close and reliable friend, at work. | .37 | .60 | .42 | .15 | **.56** | .63 | .13 | -.11 | **.79** | .65 | .15 | **.74** | -.01 | .01 |
| *3. I have let off steam by writing down my thoughts and feelings when I needed to.* | .27 | .52 | .58 | -.21 | **.84** | .59 | -.22 | **.61** | *.36* | .74 | -.16 | *.37* | **.75** | -.09 |
| 6. I have practiced breathing techniques or meditation, inside and outside of work. | .35 | .59 | .45 | .07 | **.64** | .73 | .06 | **.90** | -.18 | .81 | .10 | -.15 | **.88** | .05 |

| | | | | | | | | | | | | | | |
|---|---|---|---|---|---|---|---|---|---|---|---|---|---|---|
| *7. I have set realistic goals for my work.* | .48 | .69 | .51 | **.60** | .21 | .58 | **.58** | *.39* | -.12 | .58 | **.57** | -.15 | .21 | .24 |
| 8. I can resort to prayer according to my beliefs, whether at work or outside of it. | .39 | .62 | .45 | .14 | **.59** | .51 | .13 | **.61** | .08 | .77 | .05 | -.11 | .06 | **.86** |
| *9. I can cry if necessary, either in the hospital or outside of it.* | .36 | .60 | .52 | -.02 | **.73** | .54 | -.04 | *.32* | **.54** | .73 | -.11 | *.33* | -.11 | **.77** |
| 11. I have felt able to face problems at work. | .59 | .77 | .78 | **.89** | -.01 | .78 | **.86** | -.03 | .08 | .78 | **.88** | .11 | -.01 | -.07 |
| 12. I think I can put work problems in their proper dimension. | .58 | .76 | .81 | **.92** | -.06 | .81 | **.90** | -.06 | .06 | .81 | **.91** | .08 | -.05 | -.05 |
| 13. I have felt capable of finding solutions to problems in my work. | .60 | .77 | .81 | **.92** | -.04 | .81 | **.89** | -.01 | .04 | .81 | **.90** | .05 | -.04 | -.01 |

| | | | | |
|---|---|---|---|---|
| Proportion Total Explained | | | | |
| Variance | .43 | .58 | .66 | .73 |

*Note:* $h^2$= Communality; Λ= Factor loadings; Boldface indicates higher factor loading. Italics indicates the items with cross-loadings.

Table S3. Factor loading per item per latent factor.

| Total sample (n= 3,860) | | | | |
|---|---|---|---|---|
| | **Estimates** | **S.E.** | **C.R.** | ***p*** |
| Workload | | | | |
| fr7 | 0.80 | 0.01 | 114.42 | <.001 |
| fr11 | 0.79 | 0.01 | 105.84 | <.001 |
| Confinement | | | | |
| fr5 | 0.72 | 0.01 | 89.54 | <.001 |
| fr6 | 0.79 | 0.01 | 92.71 | <.001 |
| Loss | | | | |
| fr9 | 0.77 | 0.01 | 95.81 | <.001 |
| fr10 | 0.74 | 0.01 | 91.05 | <.001 |
| Virus exposure | | | | |
| fr2 | 0.72 | 0.01 | 66.54 | <.001 |
| fr1 | 0.61 | 0.01 | 60.40 | <.001 |
| Self-efficacy | | | | |
| fp12 | 0.86 | 0.01 | 184.20 | <.001 |
| fp13 | 0.87 | 0.01 | 188.37 | <.001 |
| fp11 | 0.86 | 0.01 | 184.37 | <.001 |
| Momentary recuperation | | | | |
| fp1 | 0.66 | 0.01 | 66.36 | <.001 |
| fp2 | 0.70 | 0.01 | 67.47 | <.001 |
| Meaning making | | | | |
| fp6 | 0.59 | 0.01 | 45.73 | <.001 |

| | | | | |
|---|---|---|---|---|
| fp8 | 0.58 | 0.01 | 45.40 | <.001 |
| Covid-19 traumatic stress | | | | |
| cs1 | 0.75 | 0.01 | 92.31 | <.001 |
| cs2 | 0.77 | 0.01 | 102.63 | <.001 |
| cs3 | 0.80 | 0.01 | 113.84 | <.001 |
| cs4 | 0.80 | 0.01 | 114.15 | <.001 |
| cs5 | 0.78 | 0.01 | 106.19 | <.001 |
| Emotional exhaustion | | | | |
| d1 | 0.78 | 0.01 | 106.56 | <.001 |
| d2 | 0.72 | 0.01 | 81.15 | <.001 |
| d3 | 0.77 | 0.01 | 100.76 | <.001 |
| d4 | 0.82 | 0.01 | 129.75 | <.001 |
| d5 | 0.74 | 0.01 | 87.33 | <.001 |
| Depersonalization | | | | |
| d6 | 0.84 | 0.01 | 112.74 | <.001 |
| d7 | 0.84 | 0.01 | 113.44 | <.001 |
| d8 | 0.70 | 0.01 | 70.58 | <.001 |
| Depersonalization | | | | |
| d9 | 0.59 | 0.01 | 49.62 | <.001 |
| d10 | 0.87 | 0.01 | 146.80 | <.001 |
| d11 | 0.77 | 0.01 | 96.36 | <.001 |
| d12 | 0.78 | 0.01 | 101.44 | <.001 |
| Generalized anxiety | | | | |
| ag1 | 0.80 | 0.01 | 126.37 | <.001 |
| ag2 | 0.84 | 0.01 | 153.50 | <.001 |

| | | | | |
|---|---|---|---|---|
| ag3 | 0.83 | 0.01 | 146.70 | <.001 |
| ag4 | 0.84 | 0.01 | 158.67 | <.001 |
| ag5 | 0.76 | 0.01 | 102.02 | <.001 |
| ag6 | 0.76 | 0.01 | 102.25 | <.001 |
| ag7 | 0.76 | 0.01 | 104.06 | <.001 |
| Depression | | | | |
| dpr1 | 0.85 | 0.01 | 160.72 | <.001 |
| dpr3 | 0.88 | 0.01 | 191.99 | <.001 |
| dpr4 | 0.75 | 0.01 | 94.20 | <.001 |
| dpr7 | 0.87 | 0.01 | 177.15 | <.001 |
| dpr6 | 0.53 | 0.01 | 43.44 | <.001 |
